# Chemical Profiling via LC‐ESI‐MS/MS and Functional Bioactivities of *Foeniculum vulgare* subsp. *Capillaceum*: Insights Into Antioxidant, Antidiabetic, and Antibacterial Potentials for Food Applications

**DOI:** 10.1111/1750-3841.70436

**Published:** 2025-07-21

**Authors:** Maroua Hadji, Hamdi Bendif, Toka Hadji, Khadidja Dehimi, Tahar Smaili, Kebaili Fethi Farouk, Ilyas Yildiz, Mohamed A. M. Ali, Ramazan Erenler, Amal Lahouaou, Fehmi Boufahja, Stefania Garzoli

**Affiliations:** ^1^ Biodiversity and Biotechnological Techniques for Plant Resources Valorization Laboratory (BTB_VRV), Department of Natural and Life Sciences, Faculty of Sciences University of M'sila, University Pole M'sila Algeria; ^2^ Biology Department, College of Science Imam Mohammad Ibn Saud Islamic University (IMSIU) Riyadh Saudi Arabia; ^3^ Laboratory of the Development and Valorization of Plant Genetic Resources, Department of Biology and Plant Ecology University of Mentouri Brothers Constantine 1 Constantine Algeria; ^4^ Department of Microbiology and Biochemistry, Faculty of Sciences University of M'sila, University Pole M'sila Algeria; ^5^ Laboratory of Microbiological Engineering and Application, Department of Biochemistry and Molecular and Cellular Biology Faculty of Nature and Life Sciences University of Mentouri Brothers Constantine 1 Constantine Algeria; ^6^ Faculty of Health Sciences and Arts, Department of Molecular Biology and Genetics Tokat Gaziosmanpaşa University Tokat Türkiye; ^7^ Application and Research Center (ALUM), Foundation of the Faculty of Health Sciences, Nutrition and Dietetics Department Iğdır University Iğdır Türkiye; ^8^ Laboratory of Plant Biotechnology and Ethnobotany, Department of Physical and Chemical Biology University of Bejaia Bejaia Algeria; ^9^ Department of Chemistry and Technologies of Drug Sapienza University Rome Italy

**Keywords:** α‐amylase inhibition, antibacterial activity, antioxidant activity, *Foeniculum vulgare* subsp. *capillaceum*, LC‐ESI‐MS/MS, phenolic compounds

## Abstract

Driven by the increasing interest in the incorporation of naturally derived ingredients in functional foods, researchers have placed greater emphasis on the process of identifying bioactive constituents from medicinal plants, as they can provide health benefits and nutritional potential. This study offers a phytochemical profiling and bioactivities investigation conducted for the first time for an Algerian wild *Foeniculum vulgare* subsp. *capillaceum*, focusing on its potential for functional food applications. Extracts from the aerial portions of the plant were prepared using a sequence of solvents, starting from *n*‐hexane, followed by acetone, methanol, and finally deionized water to efficiently separate compounds based on their solubility characteristics. Subsequently, these extracts were subjected to a detection, characterization, and precise quantification of the diverse phenolic constituents present in the samples with LC‐MS/MS. A total of 17 phenolic metabolites were characterized, with rutin and chlorogenic acid emerging as the major constituents. Among the extracts, the methanolic fraction demonstrated the greatest concentration of overall identified phenolic compounds (11.874 mg/g), accompanied by notable antioxidant capacity reflected in its effective neutralization of free radicals, recording IC₅₀ measurements of 28.69 µg/mL for DPPH radical and 24.72 µg/mL for ABTS cations. Interestingly, the hexanic extract demonstrated the strongest *α*‐amylase inhibition (50.06% at 400 µg/mL), suggesting antidiabetic potential, while the acetonic extract displayed notable antibacterial activity, particularly against *Micrococcus luteus* (MIC = 1.56 mg/mL). These findings highlight *F. vulgare* subsp. *capillaceum* as a rich reservoir of compounds with biological activities positions it as a promising addition to functional foods designed to improve nutritional value and safety.

## 1 Introduction

The genus *Foeniculum* is classified under the Apiaceae family and comprises several species that are known for their aromatic properties (Seidemann 2005). It is characterized by its perennial nature, with tall, branched stems reaching 1–2 m in height and a strong anise aroma. The lower leaves are three‐pinnate with slender, hairless linear segments, while the upper leaves are reduced. The plants lack both involucral and involucellar bracts. Its umbels typically have 6–20 rays and produce yellow flowers. The fruit is oblong, measuring 5–7 × 2–3 mm (Quezel and Santa 1963). The genus *Foeniculum* is generally considered to contain a single primary species, *Foeniculum vulgare* Mill., widely referred to as fennel. Renowned for its role in culinary traditions as a natural spice, and originally from the Mediterranean basin and Southern Europe, it is currently grown across different tropical and temperate regions globally (He and Huang 2011). Traditionally, *F. vulgare* has been historically valued across different societies for both its medicinal and culinary benefits. The seeds, leaves, and bulbs have been employed in cooking to add flavor to dishes (Tanira et al. 1996), while the seeds have also been used to make herbal teas, which is a common household remedy in Europe and Asia for treating various digestive and breathing‐related issues (Raffo, Nicoli, and Leclercq 2010). Moreover, fennel is used to relieve gas in infants. It is also consumed to improve eyesight and has been employed to promote lactation in nursing women (Rather et al. 2012). For years, *F. vulgare* has been recognized for its high content of biologically active constituents such as flavonoids, volatile oils, phenolic derivatives, and that plays a role in its distinctive aroma and therapeutic properties (He and Huang 2011). Research has shown that these compounds from *F. vulgare* exhibit various biological activities, including anti‐inflammatory, antithrombotic (Tognolini et al. 2007), antimicrobial (Kwon et al. 2002; Shahat et al. 2011), antidiabetic (El‐Soud et al. 2011), antioxidant (Ahmed et al. 2019; Faudale et al. 2008; Shahat et al. 2011), and also estrogenic effects (Albert‐Puleo 1980). These diverse properties have made fennel a subject of interest in both traditional medicine and modern pharmacological research (Ahmed et al. 2019). This species includes both cultivated and wild subspecies. The two principal forms of cultivated fennel are bitter fennel [*F. vulgare* Mill. var. *piperitum* (Ucria) Cout] and sweet fennel (*F. vulgare* Mill. var. *dulce* DC Batt. et Trab), also referred to as French or Roman fennel (*F. vulgare* Mill. var. *azoricum* Thell.), and bitter fennel, which is found both in the wild and through cultivation, is primarily grown in France, India, Japan, Germany, Italy, Romania, Hungary, southern Russia, Argentina, and the former Czechoslovakia. Sweet fennel, however, is cultivated but not found growing wild, with major growing areas including Italy, Macedonia, Bulgaria, and France (Shiva et al. 2002). Florence fennel, or finocchio, is a type of sweet fennel cultivated due to its prominent, fleshy lower stem, which is used as a vegetable (Raghavan 2006). In Arabian regions, fennel is known by several names, including Arabic: shmar, shumar, bisbas, razianj, and haba helwa (Al‐Snafi 2018). In Algeria, only three subspecies have been described by Quezel and Santa (1963), namely ssp. *piperitum*, ssp. *dulce* Mill., ssp. *capillaceum*. These subspecies are locally known as “Besbaça” or “Chbets” and are used as a food ingredient and for therapeutic purposes (Bousetla et al. 2013). *Foeniculum vulgare* subsp. *capillaceum* is a wild perennial or biennial plant with solid, non‐swollen basal stems (Quezel and Santa 1963). Based on ancestral knowledge, it has been used by local residents in many Algerian regions to relieve digestive disorders and soothe colic and respiratory ailments. The phytochemical profile of *F. vulgare* and its subspecies have been partially documented in previous studies, often focusing on its profile of volatile and aromatic compounds (Dahmani et al. 2022; Đurović et al. 2024; Milenković et al. 2022). Yet, detailed investigations into its phenolic profile, particularly in wild subspecies such as *capillaceum* native to Algeria, are still limited. *Foeniculum vulgare* subsp. *capillaceum* has been minimally studied, with the available literature consisting of only few scientific reports primarily investigating the nature and diversity of compounds within its aromatic oils (Abdellaoui et al. 2020; Hamada et al. 2021; Milenković et al. 2022; Šunić et al. 2023). Nevertheless, only one study by Marrelli et al. (2024) utilized organic solvent to determine the phenolic profile of this wild plant. This study aims to deliver the first comprehensive phytochemical and bioactivity assessment of the Algerian *F. vulgare* subsp. *capillaceum*. Through the use of LC‐ESI‐MS/MS, the phenolic composition of extracts obtained through solvents of varying polarities is characterized in detail. Quantitative evaluation of overall polyphenols and flavonoids content was conducted alongside the investigation of antioxidant potential, *α*‐amylase inhibitory activity, and antibacterial properties. By integrating advanced analytical technique with biological assays, this study brings attention to the potential of *F. vulgare* subsp. *capillaceum* as a rich natural source of functional ingredients, with promising applications in food preservation, shelf‐life extension, and the management of metabolic health through functional foods.

## 2 Material and Methods

### 2.1 Chemicals

The chemicals used throughout the current study were of the highest analytical purity and procured from established chemical suppliers Sigma‐Aldrich (Taufkirchen, Germany) and Aldrich and Honeywell including DPPH (2,2‐diphenyl‐1‐picrylhydrazyl), ABTS (2,2′‐azinobis [3‐ethylbenzoline‐6‐sulfonic acid] diammonium salt), quercetine, butylated hydroxyanisole (BHA), *α* tocopherol (TCP), Trolox (TX), acarbose, *n*‐hexane, acetone, methanol, and dimethylsulfoxide (DMSO). Seventeen phenolic standards used in LC‐ESI‐MS/MS analysis, including shikimic acid, gallic acid, chlorogenic acid, hydroxybenzaldehyde, caffeic acid, vanillin, p‐coumaric acid, salicylic acid, polydatin, trans‐ferulic acid, scutellarin, isoquercitrin, coumarin, rutin, fisetin, quercetin, and kaempferol, were purchased from Sigma‐Aldrich (Merck, Germany) with purities ≥95%.

### 2.2 Plant Material

*Foeniculum vulgare* subsp. *capillaceum* aerial parts were harvested at the flowering stage in June 2021 from the M'sila province (35°42′N 4°33′E) in Algeria. Botanical authentication was conducted by professor Smaili T., from the Department of Life and Nature Sciences at the Faculty of Sciences, Mohamed Boudiaf University, M'sila, Algeria. The identification was based on documented research (Tani et al. 2021) and the Flora of Algeria (Quezel and Santa 1963). A specimen (HM‐02) was archived within the department herbarium.

### 2.3 Extracts Preparation

In order to maximize the recovery of biologically active constituents from *F. vulgare* subsp. *capillaceum*, the collected aerial parts were meticulously cleaned, left to dry naturally at room temperature in a shaded area, and subsequently ground to a fine consistency with a mechanical grinding. In addition to deionized water, three organic solvents selected for their distinct polarities—*n*‐hexane, acetone, and methanol—were employed for the extraction process.

#### 2.3.1 Organic Extracts

The extraction procedure was modified from the protocol of Siracusa et al. (2011). The method is outlined as follows: 50 g of powdered plant material was sequentially macerated with 500 mL of solvents of increasing polarity: *n*‐hexane, then acetone, and finally methanol. Each extraction step lasted 48 h, and after each solvent treatment, the mixture was filtered and the residual plant material was used for the next solvent. Subsequently, solvent elimination was carried out using rotary evaporation at 40°C and a rotation speed of 40 RPM. The resulting extracts (HEX, ACT, MeOH) were dried using a laboratory oven and stored in amber‐colored vials at 4°C to protect them from light and degradation prior to experiments and analysis. Each extraction was conducted at standard room temperature without the application of heat, protected from light, and with occasional gentle stirring.

#### 2.3.2 Aqueous Extract

The procedure used for preparing the aqueous extract was adapted with adjustments according to the established method initially reported by Gnanaprakash et al. (2008). Precisely 50 g of finely milled plant material was transferred into 500 mL of deionized water. The blend was maintained under constant stirring while being gradually heated to 70°C, where it was held for 60 min to ensure efficient extraction of the target compounds. Afterward, the blend was stored at ambient temperature, away from light, and gently stirred from time to time over 2 days. The solution was then filtered, and filtrate elimination was carried out using rotary evaporation at 50°C and a rotation speed of 40 RPM. Finally, the resulting extract was stored in amber‐colored vials at 4°C to protect them from light and degradation prior to experiments and analysis.

### 2.4 Quantification of Phenolic Compounds Content

#### 2.4.1 Overall Polyphenols Content

The quantification of overall polyphenols was conducted by adapting the assay originally developed by Singleton and Rossi (1965), a spectrophotometric technique utilizing the Folin–Ciocalteu reagent which reacts with phenolic groups to estimate their levels. Concentrations of overall polyphenols were estimated through a reference calibration curve constructed with gallic acid and reported as µg GAE/mg E. Absorbance readings were carried out using a Multiskan SkyHigh Microplate Spectrophotometer.

#### 2.4.2 Overall Flavonoids Content

The quantification of overall flavonoids was conducted by adapting the spectrophotometric method based on aluminum chelation originally reported by Bahorun et al. (1996), a spectrophotometric technique utilizing the Folin–Ciocalteu reagent which reacts with phenolic groups to estimate their levels. Concentrations of overall flavonoids were estimated through a reference calibration curve constructed with quercetin and reported as µg QE/mg E. All spectrophotometric measurements related to the quantification assay were performed using a Multiskan SkyHigh Microplate Spectrophotometer.

### 2.5 Profiling of Extracts Using LC‐ESI‐MS/MS

The phenolic profile of *F. vulgare* subsp. *capillaceum* extracts was screened by LC‐ESI‐MS/MS analysis. The analytical method utilized in the current research was reported by Erenler et al. (2023). Chromatographic separation was performed on an Agilent Technologies Poroshell120 EC‐C18 column (100 mm × 3.0 mm, 2.7 µm) using reversed‐phase chromatography. An Agilent 1260 Infinity II liquid chromatography system coupled with a tandem mass spectrometer was employed for the characterization.

Samples were initially prepared at a concentration of 50 mg/mL of methanol, then passed through a 0.22‐µm membrane filter before introducing 5.12 µL into the LC‐MS/MS system for analysis.

The mobile phase was composed of two solvents: water (A) and methanol (B), each supplemented with 0.1% formic acid and 5 mM ammonium formate. For B mobile phase, the gradient elution was programmed so that solvent B was elevated to 25% between minute 1 and minute 3, then further increased to 50% from minute 4 through minute 12. Thereafter, the concentration of solvent B was raised to 90% during the 13‐ to 21‐min interval, before being returned to 3% for the final 22‐ to 25‐min period. The column temperature was set to 40°C throughout the analysis. Mass spectrometry settings included a capillary voltage of 4000 V, nitrogen gas flow at 11 L/min, a gas pressure of 15 psi, and a source temperature of 300°C. Method validation (covering linearity [LR], detection limits [LOD], and quantification limits [LOQ]) was performed in accordance with the criteria described by Yilmaz (2020).

### 2.6 Evaluation of in Vitro Antioxidant Activities

The antioxidant capabilities of *F. vulgare* subsp. *capillaceum* extracts were evaluated using a series of spectrophotometric assays including scavenging of DPPH and ABTS radicals, potassium ferricyanide reducing power, cupric reducing antioxidant capacity (CUPRAC), phenanthroline, and silver nanoparticles (SNP) assays. Five antioxidants standards were utilized for comparison: quercetin (QCT), ascorbic acid (AsA), *α*‐tocopherol (TCP), trolox (TX), and butylated hydroxyanisole (BHA).

#### 2.6.1 DPPH Radical Scavenging Assay

*Foeniculum vulgare* subsp. *capillaceum* extracts’ ability to *scavenge* DPPH was evaluated in line with the procedure originally described by Blois in 1958. The scavenging activity was presented as the dose‐yielding half‐maximal inhibition, reported in percentage terms (IC_50_, measured in µg/mL).

#### 2.6.2 ABTS Cation Radical Scavenging Assay

*Foeniculum vulgare* subsp. *capillaceum* extracts’ ability to scavenge ABTS was assessed according to the protocol originally reported by Re et al. (1999). The neutralizing activity was presented as the dose‐yielding half‐maximal inhibition, reported in percentage terms (IC_50_, measured in µg/mL).

#### 2.6.3 Cupric Reducing Antioxidant Capacity

*Foeniculum vulgare* subsp. *capillaceum* extracts’ capacity in reducing cupric ion was evaluated utilizing a method described by Apak et al. (2004). The antioxidant potency was presented as the absorbance value at 50% activity (A₀.₅ measured in µg/mL).

#### 2.6.4 Potassium Ferricyanide Reducing Power Assay

The power of *F. vulgare* subsp. *capillaceum* extracts in reducing ferric ions was assessed according to the protocol originally described by Oyaizu (1986). The antioxidant potency was presented as the absorbance value at 50% activity (A₀.₅ measured in µg/mL).

#### 2.6.5 Phenanthroline Assay

*Foeniculum vulgare* subsp. *capillaceum* extracts’ capacity in reducing ferric ions in phenanthroline assay was evaluated according to the protocol originally reported Szydlowskaczerniak et al. (2008). The antioxidant potency was presented as the absorbance value at 50% activity (A₀.₅ measured in µg/mL).

#### 2.6.6 Silver Nanoparticles Assay

The efficacity of *F. vulgare* subsp. *capillaceum* extracts in reducing silver ions to elemental silver, which can nucleate to form silver nanoparticles, was tested according the protocol validated by Özyürek et al. (2012). The antioxidant potency was presented as the absorbance value at 50% activity (A₀.₅ measured in µg/mL).

### 2.7 Antibacterial Activity

#### 2.7.1 Bacterial Strains

The antibacterial properties of *F. vulgare* subsp. *capillaceum* extracts were assessed against reference bacteria, namely ATCC type (American Type Culture Collection), and were sourced from the microbiology laboratory at Mohamed Boudiaf University, M'sila, Algeria. These bacteria possess pathogenic properties. Positive Gram‐bacteria included *Bacillus subtilis* ATCC9372, *Micrococcus luteus* ATCC4698, and *Staphylococcus aureus* ATCC223, and negative Gram‐bacteria included *Enterobacter cloacae* ATCC13047, *Pseudomonas aeruginosa* ATCC27853, and *Escherichia coli* ATCC25922.

#### 2.7.2 Antibacterial Susceptibility

*Foeniculum vulgare* subsp*. capillaceum* extracts’ antibacterial susceptibility was executed using the protocol of agar well diffusion, which is extensively utilized because of its simplicity and efficacy in determining the antibacterial activity (Javed et al. 2021). Sterile Mueller–Hinton agar petri plates were inoculated with bacterial suspensions (10⁸ CFU/mL), and three wells (8 mm) were made per plate. Each well contained 25 µL of *F. vulgare* subsp*. capillaceum* extract (200 mg/mL in 5% DMSO). Plates of petri were first placed at 4°C for 1 h to stabilize the medium, followed by incubation at 37°C for 24 h to promote bacterial growth to evaluate antibacterial effects. Ciprofloxacyne (CIP), vancomycine (VN), cefalexyn (CN), and imipenem (IMP) served as positive controls for the experiment. Antibacterial effect was assessed by measuring the clear zones around the wells.

#### 2.7.3 Minimum Inhibitory Concentration and Minimum Bactericidal Concentration

*F. vulgare* subsp. *capillaceum* extracts’ minimum inhibitory concentration (MIC) and minimum bactericidal concentration (MBC) are estimated according to the protocol reported by Damtie and Mekonnen (2020) with some modifications. MICs were determined using a microdilution method in 96‐well plates, with extract doses ranged between 200 and 0.78 mg/mL prepared in 5% DMSO. Gentamicin served as the positive control, tested across a doses gradient from 1.25 to 0.019 mg/mL. MICs were identified as concentrations where no growth of bacteria was visually observable. MBCs were determined by putting spots from MICs well microplates on a petri dish filled with sterile solid agar. The absence of bacteria growth on the agar was considered MBC.

### 2.8 In Vitro α‐Amylase Inhibition Activity

The activity of *F. vulgare* subsp. *capillaceum* extracts in inhibiting *α*‐amylase was assessed according the method of Lugol's iodine, as reported by Quan et al. (2019). Acarbose served as the reference compound for inhibition. The inhibition capacities of the plant extracts were presented as the percentages of inhibition, along with calculated IC₅₀ values.

### 2.9 Statistical Analysis

Each biological experiment was conducted three times using separate replicates, and the findings are reported as the arithmetic mean with the corresponding standard deviation. The IC₅₀ and A₀.₅ parameters were examined by fitting the experimental data to a linear regression model and extracting the concentration values that correspond to half‐maximal responses. Data treatment was performed using the 2016 version of XLSTAT software (developed by Addinsoft, New York, USA) to ensure rigorous statistical evaluation. To compare group differences, a one‐way analysis of variance (ANOVA) was performed, followed by Tukey's HSD test for further analysis after ANOVA results indicated significance. Statistical significance was defined at *p* < 0.05.

## 3 Results and Discussion

### 3.1 Extraction Yield

In this research, the bioactive compounds in *F. vulgare* subsp. *capillaceum* aerial parts were extracted using four solvents with increasing polarities, and yielded four crude extracts: hexanic (HEX), acetonic (ACT), methanolic (MeOH), and aqueous extracts (Aq).

The extraction yields, presented as percentages of the initial dry plant material, are displayed in Table 1.

**TABLE 1:** *F. vulgare* subsp. *capillaceum* extraction yield.

Subspecies	Extract	Yield (%)
*Foeniculum vulgare* subsp. *capillaceum*	HEX	1.13 ± 0.10c
	ACT	1.26 ± 0.21c
	MeOH	5.68 ± 0.84a
	Aq	11.90 ± 0.34b

*Note*: Categories with different letters indicate statistically significant differences.

*Foeniculum vulgare* subsp. *capillaceum* exhibited varying extraction yields depending on the solvent used. The yield from the aqueous extraction process was found to be the highest, reaching 11.90 ± 0.34%, followed by MeOH extract at 5.68 ± 0.84%. The ACT and HEX extracts had lower yields, at 1.26 ±0.21% and 1.13 ± 0.10%, respectively, with no notable difference observed between the two at *p* < 0.001.

Extraction is an important step in studying plant materials and evaluating their biological effects, as it isolates the desired soluble components while removing non‐essential substances using the right method. Additionally, plants extracts efficacy is attributed to its ability to achieve therapeutic properties, which are a direct consequence of the biological activity of the compounds contained within the extract (Sasidharan et al. 2010). The extraction method substantially impacts the amount and constituents of the resulting extract by isolating specific compounds based on their solubility and polarity (Azwanida 2015). The selection of solvents with varying polarities is a critical aspect in successfully extracting bioactive compounds from plant sources. Solvents exhibit unique polarity characteristics, and this polarity directly influences their capacity to dissolve and isolate specific classes of compounds (Kaczorová et al. 2021). Polar compounds extraction often involves the use of polar solvents, such as water and methanol; they are commonly utilized to dissolve polar compounds like phenolics, flavonoids, and glycosides, which are more soluble in such solvents. Furthermore, less polar solvents such as acetone are used to extract aglycone forms that are less polar and highly methoxylated (Dorta et al. 2011). In contrast, nonpolar solvents, such as *n*‐hexane, are commonly utilized to obtain nonpolar compounds, including lipids, terpenes, certain alkaloids, and aromatic hydrocarbons (Cravotto et al. 2022). A previous study by Nakilcioğlu‐Taş and Ötleş (2021) confirmed that solvent polarity and characteristics have an impact on phytochemical content and extraction yield. However, the increase in extraction yield could be explained by the increase of solvent polarity, since it would enhance a wide variety of compounds solubility (Muhamad et al. 2014). Our results for the methanolic extract exceeded the findings reported by Marrelli et al. (2024) for the ethanolic extract of *F. vulgare* subsp. *capillaceum*, where the yield was 2.6 ± 0.2% for the aerial parts. In contrast, Beyazen et al. (2017) obtained significantly higher yields of *F. vulgare* using methanol, a polar solvent, which yielded 24.66%, while the less polar solvent chloroform produced a lower yield of 2.81%. However, the chloroform yield still exceeds our results for the less polar solvents acetone and *n*‐hexane.

### 3.2 Estimation of Phenolic Compounds Content in Extracts

The polyphenols and flavonoids were chosen to estimate their content because they are responsible for the majority of the biological effects and properties associated with plants. The results for the total quantification of these groups within *F. vulgare* subsp. *capillaceum* extracts are presented in Table 2.

**TABLE 2:** Quantification of polyphenolic and flavonoids in *F*. *vulgare* subsp. *capillaceum* extracts.

Sample		TPC (µg GAE/mg E)	TFC (µg QE/mg E)
*F. vulgare* ssp. *Capillaceum*	HEX	71.59 ± 0.07c	41.27 ± 0.10a
	ACT	276.30 ± 0.03b	120.84 ± 0.47b
	MeOH	227.28 ± 0.71a	113.97 ± 0.31c
	Aq	92.41 ± 0.35d	45.92 ± 0.62d

*Note*: Categories with different letters indicate statistically significant differences.

Abbreviations: TFC, total flavonoids content; TPC, total polyphenols content.

According to the data in Table 2, ACT *F. vulgare* subsp. *capillaceum* extract had the maximum concentration of total polyphenols content (TPC), at approximately 276.30 ± 0.03 µg GAE/mg E, followed in order by the MeOH extract, demonstrating the second highest value of 227.28 ± 0.71 µg GAE/mg extract. The Aq and HEX extracts had a lower TPC of 92.41 ± 0.35 µg GAE/mg E and 71.59 ± 0.07 µg GAE/mg E, respectively. Regarding TFC, ACT extract also had the highest levels, with 120.84 ± 0.47 µg QE/mg E, closely followed in order by MeOH extract at 113.97 ± 0.31 µg QE/mg E. Aq extract had considerably lower TFC at 45.92 ± 0.62 µg QE/mg E, and the lowest content of TFC was in HEX extract, reaching a value of 41.27 ± 0.10 µg QE/mg E. These results confirmed that solvent polarity influences the recovery of phenolic compounds as reported in other studies (Salih et al. 2021; Xiang et al. 2024). As a matter of fact, the effectiveness and quality of the final compounds extracted from plant samples are significantly impacted by the two main factors: the type of solvent and the physicochemical traits of the sample itself (Galeotti et al. 2018). Numerous studies recommend using highly or moderately polar solvents for extracting phenolic compounds such as methanol and acetone. This is because these solvents, unlike nonpolar solvents such as *n*‐hexane, exhibit a stronger tendency to interact with phenolic compounds, resulting in more efficient extraction. Therefore, the limited extraction observed with *n*‐hexane can be attributed to the poor solubility of phenolics in its low polarity (Galanakis et al. 2011). According to the current state of knowledge, comprehensive studies quantifying the phenolic compounds content in the subspecies *F. vulgare* subsp. *capillaceum* with varying polarities are currently limited. Our results were superior to those of Beyazen et al. (2017) who confirmed the lower total polyphenols and flavonoids contents in the chloroform extract of *F. vulgare*, and higher contents in hydromethanolic and methanolic extracts.

### 3.3 Phenolic Profile Analysis

LC‐MS/MS techniques are highly effective for investigating the complex variety of plant metabolites, which frequently consist of diverse polar, semi‐polar secondary compounds (El Sayed et al. 2020).

**FIGURE 1:**
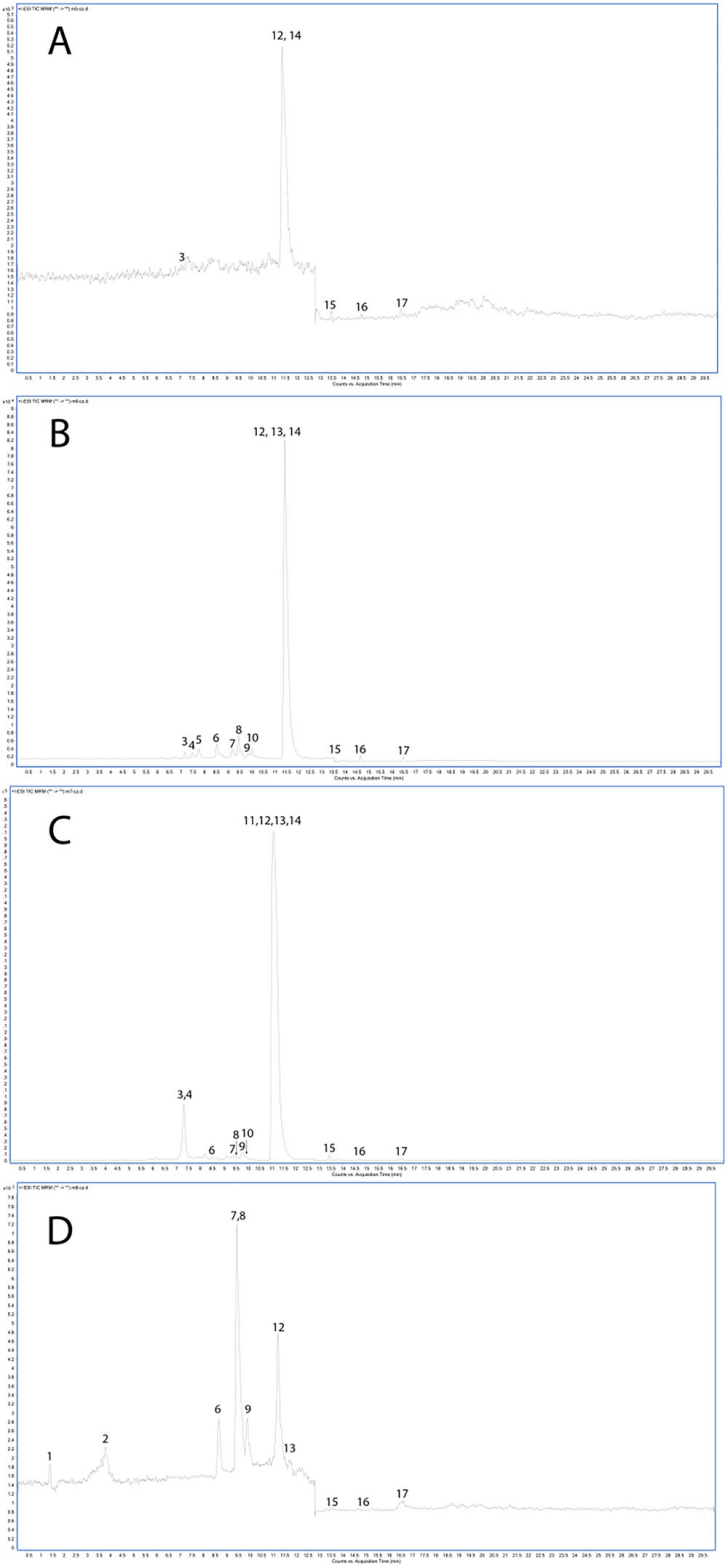
TIC HPLC‐MS/MS chromatographic profiles of *F. vulgare* subsp. *capillaceum* extracts (A: hexanic extract, B: aceonic extract, C: methanolic extract, and D: aqueous extract).

The phenolic profile of *F. vulgare* subsp. *capillaceum* areal parts extracts was comprehensively characterized through LC‐ESI‐MS/MS analysis, as depicted in Figure 1 and reported in Table 4. A total of 17 phenolic compounds were successfully characterized within the extracts. Their identification was achieved through a comprehensive analysis of their unique MS fragmentation patterns, accurate high‐resolution mass measurements, and characteristic retention times, as summarized in Table 3. Subsequent to identification, the quantified levels of each compound were determined. Table 4

**TABLE 3:** Analytical parameters of identified compounds in *F. vulgare* subsp. *capillaceum* extracts by LC‐MS/MS.

No.	Compound	Retention time (min)	Ion mode and reported in Table 4	Ion source	Ion transition	*R* ^2^	LR (µg/L)	Calibration equations	LOD (µg/L)	LOQ (µg/L)
1	Shikimic acid	1.453	Neg.	ESI	172.0→92.9	0.9976	1251–20,000	*y* = 521.37*x* + 3102.5	150.69	500.39
2	Gallic acid	3.769	Neg.	ESI	170.0 → 124.9	0.9969	31.25–500	*y* = 9824.6*x* + 275.9	71.674	185.862
3	Chlorogenic acid	7.326	Neg.	ESI	342.0 → 181.0	0.9993	31.25–500	*y* = 11,052*x* + 197.3	11.589	259.023
4	Hydroxybenzaldeyde	7.618	Neg.	ESI	121.0 → 92.0	0.9993	15.625–250	*y* = 14,536*x* + 158.8	49.742	128.651
5	Caffeic acid	7.828	Neg.	ESI	187.9 → 153.1	0.9994	31.25–500	*y* = 9482.3*x* + 312.7	69.205	24.162
6	Vanillin	8.506	Pos.	ESI	153.0 → 125.0	0.9949	62,5–1000	*y* = 8034.6*x* + 874.2	145.885	405.411
7	*p*‐Coumaric acid	9.581	Neg.	ESI	173.0 → 119.0	0.9987	31.25–500	*y* = 10,235*x* + 229.9	35.348	175.416
8	Salicylic acid	9.503	Neg.	ESI	137.0 → 93.1	0.9981	112.5–1800	*y* = 8935.2*x* + 305.6	476.695	829.646
9	Polydatin	9.764	Pos.	ESI	390.9 → 228.9	0.9987	7.8125–125	*y* = 11,453*x* + 120.1	11.471	18.411
10	Trans‐ferulic acid	10.088	Neg.	ESI	182.1 → 133.9	0.995	31.25–1000	*y* = 9563.1*x* + 214.6	61.184	115.276
11	Scutellarin	11.179	Pos.	ESI	462.8 → 286.8	0.9978	9.375–300	*y* = 12,786*x* + 342.7	31.346	40.013
12	Isoquercitrin	11.461	Pos.	ESI	464.9 →302.8	0.9982	18.75–300	*y* = 11,231*x* + 279.5	99.382	11.268
13	Coumarin	11.643	Pos.	ESI	183.1 → 144.9	0.9978	31.27–1000	*y* = 7832.5*x* + 158.6	129.23	50.245
14	Rutin	11.687	Pos.	ESI	611.0 → 302.8	0.998	125–2000	*y* = 13,467*x* + 452.9	595.597	240.672
15	Fisetin	13.420	Pos.	ESI	287.0 → 137.0	0.9954	15.625–250	*y* = 10,853*x* + 347.2	108.961	443.662
16	Quercetin	14.799	Neg.	ESI	300.8 →151.0	0.9964	27.4–440	*y* = 11,653*x* + 238.4	169.129	46.555
17	Kaempferol	16.546	Neg.	ESI	284.9 → 116.9	0.9997	312.5–10,000	*y* = 9312.7*x* + 384.6	18.683	54.004

Abbreviations: Neg, negative; Pos, positive.

**TABLE 4:** Phenolic Profile of *F. vulgare* subsp. *capillaceum* determined by LC‐MS/MS.

N o.	Compound	HEX	ACT	MeOH	Aq
		Compound levels (mg/g)		
1	Shikimic acid	ND	ND	ND	0.255
2	Gallic acid	ND	ND	ND	0.127
3	Chlorogenic acid	0.016	0.067	3.006	ND
4	Hydroxybenzaldeyde	ND	0.032	0.008	ND
5	Caffeic acid	ND	0.008	0.029	ND
6	Vanillin	ND	0.004	0.010	0.012
7	*p*‐Coumaric acid	ND	0.013	0.031	0.049
8	Salicylic acid	ND	0.018	0.060	0.035
9	Polydatin	ND	0.002	0.007	0.003
10	Trans‐ferulic acid	ND	0.053	0.144	ND
11	Scutellarin	ND	ND	0.014	ND
12	Isoquercitrin	0.009	0.004	0.011	0.007
13	Coumarin	ND	0.026	0.391	0.077
14	Rutin	0.036	0.356	8.142	ND
15	Fisetin	0.002	0.002	0.002	0.002
16	Quercetin	0.006	0.006	0.006	0.006
17	Kaempferol	0.012	0.014	0.012	0.013
Total quantification (mg/g)		0.193	0.603	11.874	0.585

Abbreviation: ND, not identified.

The MeOH extract displayed the highest accumulation of identified phenolic compounds at a concentration of 11.887 mg/g. Subsequently, the next accumulation of identified phenolic compounds was observed in the ACT extract at 0.607 mg/g, the Aq extract at 0.342 mg/g, and finally, the HEX extract at 0.193 mg/g. Rutin emerged as the predominant phenolic constituent in the methanolic extract, reaching a maximum concentration of 8.142 mg/g. Chlorogenic acid was the second most prevalent compound in the same extract, present at a concentration of 3.006 mg/g. The remaining identified phenolic compounds were observed at varying levels, with none exceeding a concentration of 1 mg/g.

Through LC‐ESI‐MS/MS findings, the analysis revealed that the plant extracts comprised a variety of phenolic constituents, with their distribution and solubility differing based on the solvent applied during extraction.

In the Aq extract, shikimic acid and gallic acid were uniquely identified, present at 0.255 and 0.127 mg/g, respectively. Chlorogenic acid was present in all extracts except for the Aq one, with the highest concentration observed in the MeOH reaching 3.006 mg/g. Subsequently, lower concentrations were found in the ACT extract at 0.067 mg/g and the HEX extract at 0.016 mg/g. Hydroxybenzaldehyde was exclusively found in the ACT extract at a concentration of 0.032 mg/g, which was also identified within the MeOH extract at a concentration of 0.008 mg/g extracts. Vanillin was identified in the ACT extract at a concentration of 0.004 mg/g and was also present within the MeOH extract at a concentration of 0.010 mg/g. However, the highest concentration of vanillin was observed in the Aq extract, reaching a level of 0.012 mg/g. *p*‐Coumaric acid was detected in the ACT extract at a concentration of 0.013 mg/g and in the MeOH extract at a concentration of 0.031 mg/g. Notably, the highest concentration of *p*‐coumaric acid was observed within the Aq extract, reaching 0.049 mg/g. Salicylic acid demonstrated a differential distribution across the extracts, with the highest concentration observed in the MeOH extract at 0.060 mg/g, followed by lower levels in the Aq extract at 0.035 mg/g and the ACT extract at 0.018 mg/g. Moreover, the MeOH extract demonstrated the most effective extraction of polydatin compared to the other solvents, yielding a concentration of 0.007 mg/g, while the ACT and Aq extracts yielded 0.002 and 0.003 mg/g, respectively. Also, the MeOH extract exhibited a significantly higher concentration of trans‐ferulic acid, reaching 0.144 mg/g, compared to the ACT extract, which reached 0.053 mg/g. Scutellarin was exclusively detected within the MeOH extract at a concentration of 0.014 mg/g. Isoquercitrin was consistently present across all extracts, with concentrations of 0.009 mg/g in the HEX extract, 0.004 mg/g in the ACT extract, 0.011 mg/g in the MeOH extract, and 0.007 mg/g in the Aq extract. The MeOH extract displayed the highest concentration of coumarin at 0.391 mg/g, while lower levels were observed in the ACT extract at 0.026 mg/g and the Aq extract at 0.077 mg/g. Rutin was predominantly found in the MeOH extract at a concentration of 8.142 mg/g, with lower levels detected in the ACT extract at 0.356 mg/g and the HEX extract at 0.036 mg/g. Fisetin appeared uniformly in every extract analyzed, maintaining a steady level of 0.002 mg/g, while quercetin was present in all extracts at a concentration of 0.006 mg/g. Kaempferol exhibited slight variations in the concentration across the extracts. Higher levels were observed in the ACT extract at 0.014 mg/g and the Aq extract at 0.013 mg/g compared to the HEX extract at 0.012 mg/g and the MeOH extract at 0.012 mg/g.

When compared to the findings from previous studies, the flavonoid and phenolic acid profile of *F. vulgare* subsp. *capillaceum* extracts shows significant similarities with the findings from various parts of the *F. vulgare* species. Similarly, Salami et al. (2016) identified *p*‐coumaric acid, caffeic acid rutin, chlorogenic acid, and quercetin in methanolic extracts from 23 fennel samples. Moreover, the detection of gallic acid, quercetin, and salicylic acid in *F. vulgare* subsp. *capillaceum* aerial parts aqueous extract is in agreement with the findings of Sayah et al. (2020), who identified these compounds in aqueous extracts from both the rootstock and leaves of fennel (*F. vulgare*). Gore et al. (2021) mentioned in their research that vanillin is present in *F. vulgare*. An investigation had been conducted into the phenolic profile of wild *F. vulgare* Mill. populations native to Tunisia, focusing on methanolic extracts of the seeds. The study identified the presence of quercetin, rutin, chlorogenic acid, caffeic acid, and trans‐ferulic acid. In addition, Akbari et al. (2023) identified salicylic acid, caffeic acid, and chlorogenic acid as the predominant phenolic components in methanolic seed extracts from three fennel genotypes. Our research detected all these compounds in the aerial parts of *F. vulgare* ssp. *capillaceum*, thereby suggesting the presence of these phenolic acids across different parts of the plant. However, polydatin, scutellarin, fisetin, and hydrobenzaldhyde are identified in the species *F. vulgare* for the first time in the current study.

Building on these findings, it is important to note that due to the examination of different plant parts, a comprehensive comparison of all phenolic compounds across studies was not possible. Consistent with previous research, chlorogenic and caffeic acids have been repeatedly identified as the primary phenolic acids in fennel, while quercetin and rutin have been recognized as the major flavonoids.

The quantitative and qualitative variations of compounds between extracts can be attributed to the differences in solvents polarities, which affected the solubility of these compounds differently (Muhamad et al. 2014). All identified compounds have been extensively documented in the scientific literature for their various biological activities. However, ongoing research continues to reveal novel aspects of their biological and beneficial effects, expanding the understanding of their potential in fields such as human health, agriculture, and environmental sustainability. Indeed, rutin and chlorogenic acid were identified as the predominant compounds in *F. vulgare* subsp. *capillaceum* aerial parts. Rutin is a well‐known flavonoid that exhibits strong antioxidant and anti‐inflammatory properties (Akash et al. 2024; Tian et al. 2020) and has demonstrated promising cardioprotective effects (Z. Wang et al. 2022). Chlorogenic acid is an antioxidant known for its benefits to metabolic health and cardiovascular function (Nguyen et al. 2024). Shikimic acid, a key intermediate in aromatic amino acid biosynthesis, has recently been implicated in antiviral activities (Xin et al. 2025). Gallic acid, a well‐known antioxidant, exhibits anti‐inflammatory properties (Kahkeshani et al. 2019). Hydroxybenzaldehyde demonstrated antimicrobial and anticancer activities (Lim et al. 2008), while caffeic acid exhibits strong hepatoprotective and antioxidant effects (Wu et al. 2024; Zhu et al. 2024). Vanillin, known for its aromatic properties, also possesses antioxidant and antimicrobial activities (Bezerra et al. 2017). *p*‐Coumaric acid exhibited antioxidant properties and may offer protection against oxidative stress‐related diseases (Godarzi et al. 2020). Salicylic acid is a well‐known anti‐fungal agent (Gacnik et al. 2023). Polydatin, a resveratrol precursor, exhibited antioxidant, cardioprotective, and neuroprotective properties (Karami et al. 2022). Trans‐ferulic acid has shown strong antioxidant effects, making it a good choice for some nanotechnology applications (Trombino et al. 2013). Scutellarin is recognized for its neuroprotective effects, particularly in cerebrovascular diseases (C. Wang et al. 2023). Isoquercitrin aids in hair growth and helps improve diabetic nephropathy (Manzoor et al. 2024; Zhang et al. 2024). Coumarin is noted for its anticoagulant, antimicrobial, and anti‐tumor properties (Akkol et al. 2020). The presence of flavonoids such as fisetin, quercetin, and kaempferol further supports the potential therapeutic benefits of the plant. Fisetin exhibits anti‐aging properties (R. Zhao et al. 2023), while quercetin possesses antioxidant, anti‐inflammatory, and anti‐cancer activities (Lotfi et al. 2023). Kaempferol is associated with neuroprotective and cardioprotective activities (Kamisah et al. 2023). The isolation and purification of these bioactive molecules, combined with their evaluation natural product chemistry, position these compounds as promising candidates for the development of novel therapeutic agents, underscoring the enduring significance of natural sources in drug discovery.

### 3.4 Antioxidant Activity

Table 5 exhibits the results of antioxidant activities of *F. vulgare* subsp. *capillaceum* extracts, which are compared to antioxidant standards (QCT, BHA, TCP, Trolox, and AsA) and expressed in terms of IC_50_ and A_0.50_.

**TABLE 5:** In vitro antioxidant power of *F. vulgare* subsp. *capillaceum* extracts.

Samples		DPPH	ABTS	CUPRAC	Phenanthroline	Potassium ferricyanide RP	SNP
		(IC_50_ µg/mL)		(A_0.5_ µg/mL)			
*F. vulgare* subsp. *capillaceum*	HEX	112.33 ± 0.09c	60.43 ± 0.09b	169.58 ± 0.29b	>200	131.77 ± 0.18c	190.59 ± 0.43a
	ACT	41.51 ± 0.58e	36.07 ± 0.16c	33.46 ± 0.11e	81.16 ± 0.32c	59.00 ± 1.22d	156.86 ± 0.86c
	MeOH	28.69 ± 0.92g	24.72 ± 0.76e	32.40 ± 0.17e	63.19 ± 0.84b	42.34 ± 0.91a	89.69 ± 1.09b
	Aq	196.68 ± 0.47a	90.02 ± 1.09a	80.07 ± 0.41d	>200	178.06 ± 1.27b	>200
Standards	QCT	3.63 ± 0.18b	<1.5625	2.12 ± 0.04c	5.40 ± 0.20e	1.01 ± 0.24e	–
	BHA	9.69 ± 0.18f	8.01 ± 0.11d	–	1.84 ± 0.12a	1.68 ± 0.37e	–
	TCP	6.75 ± 0.06d	–	17.00 ± 0.37a	7.69 ± 1.39e	3.46 ± 0.06f	–
	TX	–	–	–	–	–	34.17 ± 1.23d
	AsA	–	–	–	–	–	7.14 ± 0.05e

*Note*: Categories with different letters indicate statistically significant differences.

The antioxidant activity of *F. vulgare* subsp. *capillaceum* extracts was evaluated using multiple methods, including DPPH, ABTS, CUPRAC, phenanthroline, and potassium ferricyanide assays. Across all the methods, the MeOH extract consistently exhibited the highest antioxidant capacity, followed by the ACT extract, while the HEX and Aq extracts demonstrated weaker activities.

In the DPPH assay, the MeOH extract showed the strongest radical scavenging activity (IC₅₀ = 28.69 ± 0.92 µg/mL), followed by ACT (IC₅₀ = 41.51 ± 0.58 µg/mL), HEX (IC₅₀ = 112.33 ± 0.09 µg/mL), and Aq (IC₅₀ = 196.68 ± 0.47 µg/mL). Similar trends were observed in the ABTS assay, where the MeOH and ACT extracts displayed strong activities (IC₅₀ = 24.72 ± 0.76 µg/mL and 36.07 ± 0.16 µg/mL, respectively), whereas the HEX and Aq extracts were markedly less effective. The results from CUPRAC, phenanthroline, and potassium ferricyanide assays further confirmed the superior antioxidant potential of the MeOH and ACT extracts, with A₀.₅ values ranging between 32.40 and 63.19 µg/mL. In contrast, HEX and Aq extracts consistently showed weaker reduction capabilities, with A₀.₅ values exceeding 130 µg/mL in most assays. Additionally, the silver nanoparticle assay also confirmed the stronger reduction activity of the MeOH extract (A₀.₅ = 89.69 ± 1.09 µg/mL) compared to the other extracts. These results collectively highlight a consistent antioxidant pattern across different methods, with methanolic extract exhibiting the highest efficacy. A clear correlation was observed between the antioxidant potential and the total polyphenol content of the extracts. Extracts rich in phenolic compounds, particularly the MeOH and ACT fractions, displayed stronger radical scavenging and reducing power. Conversely, HEX and Aq extracts, with relatively lower polyphenol levels, demonstrated weaker antioxidant effects. This dose–activity relationship mirrors the findings from D. Zhao et al. (2024), who reported that *Emilia prenanthoidea* extracts with higher flavonoid content exhibited stronger antioxidant activity, while samples with lower flavonoid levels still showed a noticeable correlation between compound content and antioxidant performance. Moreover, the identification of phenolic compounds with antioxidant properties, such as rutin and chlorogenic acid, by LC‐ESI‐MS/MS analysis highlights their significant contribution to the antioxidant potential of *F. vulgare* subsp. *capillaceum* extracts, especially in the methanolic extract. Numerous studies have documented their strong antioxidant characteristics (Choi et al. 2021; Liang and Kitts 2015). These activities are attributed to the five active hydroxyl groups in chlorogenic acid, which interact with free hydroxyl radicals, and to the presence of a catechol group on the B‐ring and a free hydroxyl group at C3 in rutin molecule, which are capable of binding transition metal ions (Lafay et al. 2006; Pivec et al. 2019). All other identified compounds in *vulgare* subsp. *capillaceum* were mentioned in previous scientific literature for their antioxidant effect, including catechin (Grzesik et al. 2017), scutellarein, isoquercitrin (X. Li et al. 2016), fisetin (Sip et al. 2024), kaempferol (Chen et al. 2013), vanillin (Oladimeji et al. 2022), polydatin and resveratrol (Z. Li et al. 2021), caffeic acid (Spagnol et al. 2019), *p*‐coumaric acid (Shen et al. 2019), salicylic acid (R. S. Borges et al. 2014), trans‐ferulic acid (Rezaeiroshan et al. 2021), and quercetin (W. Li et al. 2019). Moreover, polyphenols compounds in the extracts have the capacity to function separately or in synergy with other compounds (Mitra et al. 2022).

Based on our current knowledge, this is the first study to comprehensively investigate the free radical scavenging and metal ion reducing capacities of *F. vulgare* subsp. *capillaceum* using extracts with varying polarities. While one study reported the antioxidant activity of *F. vulgare* subsp. *capillaceum* aerial parts using an ethanolic extract and the DPPH assay (Marrelli et al. 2024), our results for the DPPH assay of the polar extract MeOH demonstrated higher antioxidant activity compared to the ethanolic extract in this study, which exhibited an IC_50_ value of 293.13 ± 22.98 µg/mL. Moreover, an investigation was conducted into the antioxidant properties of methanolic extracts from 16 samples of wild of *F. vulgare* Mill. native to Tunisia, using the DPPH assay. The lowest IC_50_ value was found in the MeOH extract of *F. vulgare* Mill. grown in the Beni Ayech region, which was 23.66 ± 0.66 µg/mL and is similar to the value of the MeOH extract in the current study. The remaining samples exhibited IC_50_ values lower than our finding, which reached 975.66 ± 0.66 µg/mL. Also, El Ouariachi et al. (2014) reported the scavenging activity of two extracts from *F. vulgare* leaves, one nonpolar (diethyl ether) and one moderately polar (ethyl acetate), with results significantly exceeding ours at 6.2 and 1.5 µg/mL, respectively.

### 3.5 Alpha‐Amylase Inhibition Activity

The results of alpha amylase inhibition activity of *F. vulgare* subsp. *capillaceum* extracts are presented in Table 6.

**TABLE 6:** *α*‐Amylase inhibition activity of *F. vulgare* subsp. *capillaceum* extracts.

		400 (µg/mL)	200 (µg/mL)	100 (µg/mL)	IC_50_ (µg/mL)
*F. vulgare* subsp. *capillaceum*	HEX	50.06 ± 0.08c	19.39 ± 0.92a	10.73 ± 0.66d	393.12 ± 0.88a
	ACT	40.70 ± 0.43d	17.51 ± 0.12c	7.01 ± 0.22b	>400
	MeOH	13.67 ± 0.09e	8.10 ± 0.18d	3.26 ± 0.19b	>400
	Aq	10.37 ± 0.34b	7.34 ± 0.35e	5.36 ± 0.12a	>400
Acarbose		53.05 ± 1.59a	37.21 ± 3.54b	31.81 ± 2.89e	365.93 ± 1.70a

*Note*: Categories with different letters indicate statistically significant differences.

Among the extracts tested, the HEX extract exhibited the strongest inhibitory activity, reaching a maximum of 50.06 ± 0.08% at a concentration of 400 µg/mL. The ACT extract showed an inhibition of 40.70 ± 0.43% at the same concentration. In contrast, the MeOH and Aq extracts displayed significantly lower inhibitory effects, reaching only 13.67 ± 0.09% and 10.37 ± 0.34% inhibition, respectively, at 400 µg/mL. The IC_50_ value of the HEX extract was determined to be 393.12 ± 0.88 µg/mL, close to the IC_50_ value of acarbose, which was 365.93 ± 1.70 µg/mL.

Based on the inhibition results of *F. vulgare* subsp. *capillaceum* extracts, it is evident that the inhibition activity increases with a decrease in solvent polarity, with nonpolar solvent being the most effective, followed by moderately polar solvent.

Numerous studies investigating plant extracts for *α*‐amylase inhibition have employed solvents with varying polarities, including *n*‐hexane. These studies frequently report *n*‐hexane extracts as exhibiting the strongest inhibitory activity against *α*‐amylase (Mogole et al. 2020; Vadivelan et al. 2018). This observation aligns with the fact that *n*‐hexane, a nonpolar solvent, effectively extracts compounds with low polarity, including terpenoids structures, lipids, and aromatic hydrocarbons which work as alpha amylase inhibitors (Cravotto et al. 2022; Gong et al. 2020). Additionally, phenolic compounds are widely recognized as a key source of *α*‐amylase inhibitors (Ćorković et al. 2022), including quercetin, isoquercetin, and chlorogenic acid, which were identified in the *F. vulgare* subsp. *capillaceum* aerial parts hexanic extract, in the current study (Aleixandre et al. 2021; Y. Li et al. 2009).

To the best of our knowledge, this research is the first to report *α*‐amylase inhibitory properties of *F. vulgare* subsp. *capillaceum* aerial parts. However, a previous study by Godavari et al. (2018) showed that the *n*‐butanolic extract of *F. vulgare* seeds was more effective in inhibiting *α*‐amylase than the ethyl acetate extract, which is more polar than *n*‐butanol. This is similar to our findings, where the less polar extracts proved to be more effective.

### 3.6 Antibacterial Activity

The inhibition zone diameter for each extract, as well as the positive and negative controls, is presented in Table 7. Additionally, the MIC and MBC values are presented in Tables 8 and 9, respectively.

**TABLE 7:** Antibacterial activity of *F. vulgare* subsp. *capillaceum* extracts using the agar diffusion method.

Bacterial strains		*B. subtilis*	*S. aureus*	*E. cloacae*	*E. Coli*	*P*. *aeruginosa*	*M. luteus*
Inhibition zone diameter (mm)							
*F. vulgare* ssp. *capillaceum*	HEX	10 ± 00c	15 ± 00b	NA	NA	10 ± 0d	NA
	ACT	14 ± 00b	12 ± 00d	14 ± 00b	NA	16 ± 00b	14 ± 00b
	MeOH	NA	12 ± 00d	NA	NA	16 ± 00b	NA
	Aq	NA	NA	NA	NA	NA	NA
Positive control	VN	15 ± 00a	NT	NT	NT	NT	16 ± 00a
	CIP	NT	NT	NT	NT	22 ± 0a	NT
	CN	NT	NT	18 ± 00a	NT	NT	NT
	IMP	NT	16 ± 00a	NT	20 ± 00	NT	NT
Negative control	DMSO 5%	NA	NA	NA	NA	NA	NA

*Note*: Categories with different letters indicate statistically significant differences.

Abbreviations: NA, no activity; NT, not tested.

**TABLE 8:** Minimum inhibitory concentrations of and *F. vulgare* subsp. *capillaceum* extracts.

Bacterial strains		*B. subtilis*	*S. aureus*	*E. cloacae*	*P. aeruginosa*	*M. luteus*
	MIC (mg/mL)					
***F. vulgare* ssp. *capillaceum***	HEX	50 ± 0.00	25 ± 0.00	–	50 ± 0.00	–
	ACT	3.125 ± 0.00	12.5 ± 0.00	12.5 ± 0.00	3.125 ± 0.00	1.56 ± 0.00
	MeOH	–	12.5 ± 0.00	–	3.125 ± 0.00	–
**G**		<0.019	<0.019	<0.019	<0.019	<0.019

**TABLE 9:** Minimum bactericidal concentrations of *F. vulgare* subsp. *capillaceum* extracts.

Bacterial strains		*B. subtilis*	*S. aureus*	*E. cloacae*	*P. aeruginosa*	*M. luteus*
	MBC (mg/mL)					
*F. vulgare* ssp. *capillaceum*	HEX	100 ± 0.00	50	–	100 ± 0.00	–
	ACT	6.25 ± 0.00	25 ± 0.00	25 ± 0.00	6.25 ± 0.00	3.125 ± 0.00
	MeOH	–	25 ± 0.00	–	6.25 ± 0.00	‐

The antibacterial activity of *F. vulgare* ssp. *capillaceum* extracts varied depending on the bacterial strain. Furthermore, ACT extract was effective against most of the bacterial strains. It exhibited antibacterial activity, with the greatest inhibition recorded against *P. aeruginosa* (16 ± 00 mm), followed by *E. cloacae*, *B. subtilis* and *M. luteus* (14 ± 00 mm), while also showing notable activity against *S. aureus* (12 ± 00 mm), and no inhibition was detected for *E. coli*.

MeOH extract demonstrated the strongest effect against *P. aeruginosa* with an inhibition zone of 16 ± 00 mm, and moderate action against *S. aureus* (12 ± 00 mm). However, it was inactive against *E. cloacae*, *B. subtilis*, *M. luteus*, and *E. coli*. HEX extract showed moderate efficacy, with the largest inhibition zone observed against *S. aureus* (15 ± 00 mm), followed by lower effects on *P. aeruginosa* and *B. subtilis* (both 10 ± 00 mm). However, no inhibition was detected for *E. cloacae*, *E. coli*, or *M. luteus*. In addition to 5% DMSO, the aqueous extract did not display any antibacterial activity. Also, all extracts had a lower inhibition zone diameters compared with the antibiotic results (Table 7).

Based on the MIC and MBC values for *F. vulgare* ssp. *capillaceum* extracts, significant variations in antibacterial efficacy were noted among the different bacterial strains tested. HEX extract showed an MIC of 50 mg/mL against *P. aeruginosa* and *B. subtilis*, while demonstrating higher activity for *S. aureus* with an MIC of 25 mg/mL. The ACT extract displayed superior inhibiting activity, particularly against *M. luteus* (MIC: 1.56 mg/mL), *B. subtilis*, and *P. aeruginosa* (MIC: 3.125 mg/mL), while showing a significantly lower MIC for *S. aureus* and *E. cloacae* (12.5 mg/mL). The MeOH extract inhibited *P. aeruginosa* and *S. aureus* at MIC values of 3.125 and 12.5 mg/mL, respectively. The extracts exhibited lower MICs when compared to gentamicin, the reference antibiotic. Gentamicin demonstrated a significantly higher MIC against all bacterial strains, which is lower than 0.019 mg/mL.

For the MBC, HEX extract exhibited an MBC of 100 mg/mL for *B. subtilis* and *P*. aeruginosa, and 50 mg/mL for *S. aureus*. Meanwhile, the ACT extract demonstrated much higher bactericidal activity, particularly against *M. luteus* (3.125 mg/mL), *B. subtilis*, and P. *aeruginosa* (6.25 mg/mL). Also, the ACT extract was effective against *S. aureus* and *E. cloacae* with MBC of 25 mg/mL. The MeOH extract presented the highest MBC of 6.25 mg/mL for *P. aeruginosa*. It was also effective in achieving the MBC for *S. aureus* at 25 mg/mL.

The MIC and MBC findings in our study confirmed the results obtained using well agar diffusion assay and indicate that the ACT extract is the most effective extract anti‐gram positive and negative bacteria, expect for *E. coli*. This suggests that ACT may yielded a higher concentration of antibacterial phytochemicals from *F. vulgare* subsp*. capillaceum* aerial parts with greater activity against the tested bacterial strains in comparison to *n*‐hexane, methanol, and water. Most of the identified phenolic compounds and *F. vulgare* ssp. *capillaceum* extracts are known for their capacity to suppress and kill both Gram‐positive and Gram‐negative bacteria at different concentrations (Khameneh et al. 2019; Lobiuc et al. 2023; Madkour et al. 2024). Their effectiveness depends on the type of bacteria, the strain, and the chemical structure of the compounds. These compounds damage bacterial cell walls and membranes, causing the contents to leak out. They also block important enzymes and disrupt protein and DNA production, which leads to the death of the bacteria (Lobiuc et al. 2023). Chlorogenic acid, an ester of caffeic and quinic acids, exerts membrane‐disruptive effects, triggers oxidative damage, and impairs bacterial metabolic enzymes (Lou et al. 2011), while rutin is a glycosylated flavonol and is known for its bacteriostatic and bactericidal actions, primarily via the inhibition of DNA gyrase, interference with cell division, and alteration of membrane permeability (Górniak et al. 2018). Interestingly, both methanolic and acetone extracts exhibited a similar phenolic profile, with rutin and chlorogenic acid identified as their major constituents. However, the superior performance of ACT suggests that phenolic efficacy is not solely concentration‐dependent, but also influenced by solvent polarity, compound interactions, and extractability of synergistic minor constituents that may co‐elute with active phenolics. According to Eloff (1998), acetone is a semi‐polar solvent that often extracts smaller and more lipophilic phenolics which may not have been identified in our study but are capable of better penetrating bacterial membranes, especially in Gram‐negative bacteria like *P. aeruginosa* and *E. cloacae*, which possess complex outer membranes.

Furthermore, the antibacterial activity of phenolic compounds in general is largely influenced by their chemical structure. Variations in the number and position of hydroxyl and methoxy groups, as well as their overall molecular shape and degree of polymerization, affect how these compounds interact with bacterial membranes. Generally, flavonoids with fewer hydroxyl groups on their B ring tend to be more hydrophobic, which enhances their ability to insert into and disrupt microbial lipid bilayers, particularly in Gram‐negative bacteria. However, even highly hydroxylated flavonoids can exert strong antimicrobial effects, as their interaction with membrane components may lead to structural disintegration or oxidative stress within the bacterial cell. These structural features may also interfere with the expression or secretion of bacterial toxins and enzymes, thereby reducing virulence. Altogether, such mechanisms demonstrate the versatile ways in which flavonoids can inhibit bacterial growth and support their potential as natural alternatives to conventional antimicrobials (Górniak et al. 2018). Moreover, many flavonoids are recognized for their strong antibiofilm effects. They have shown the ability to reduce biofilm formation in *Staphylococcus aureus*. These effects are attributed to their interference with bacterial adhesion and early biofilm structuring. Kaempferol and quercetin have been identified as the inhibitors of quorum sensing which is the bacterial communication system that regulates biofilm formation (De La Fuente‐Núñez et al. 2012; Vikram et al. 2010). Quercetin, for instance, disrupts the production of extracellular polymers such as alginate, interferes with bacterial adherence, and modulates iron metabolism, a critical factor in *Pseudomonas aeruginosa* biofilm growth (Ouyang et al. 2016).

Many studies have consistently supported our findings, demonstrating that acetone is particularly effective in extracting antibacterial compounds from various plant sources, surpassing the extraction efficiency of other solvents such as methanol, chloroform, and hexane (A. Borges et al. 2020; Eloff 1998). Kaur and Arora (2009) found that acetone was the most efficient solvent for extracting bioactive compounds with antibacterial properties from *F. vulgare* seeds. Their study demonstrated that the acetone extract inhibited *S. aureus*, *P. aeruginosa*, and *E. coli* more effectively than hexane and ethanol extracts, with MIC values of 5, 5, and 10 mg/mL against these strains, respectively. Another study by Erdoğan Eliuz (2023) reported that *Escherichia coli* was resistant to the methanolic extract from *F. vulgare*. However, the methanolic extract exhibited an MIC of 100 mg/mL against *S. aureus*, which is lower than our findings.

## 4 Conclusion

This study provides the first comprehensive characterization of the phenolic profile of Algerian *F. vulgare *subsp*. capillaceum* aerial parts using LC‐ESI‐MS/MS, alongside an evaluation of its biological activities. Four extracts (hexane, acetone, methanol, and aqueous) were prepared, revealing significant variations in phytochemical composition and bioactivity. The acetonic extract contained the highest levels of polyphenols and flavonoids, while the methanolic extract demonstrated superior antioxidant capacity, with IC_50_ values of 28.69 µg/mL for DPPH and 24.72 µg/mL for ABTS. The hexanic extract exhibited moderate *α*‐amylase inhibition (50.06% at 400 µg/mL), suggesting potential applications in managing blood sugar levels. Additionally, the acetonic extract showed notable antibacterial activity, particularly against *M. luteus*, with a MIC of 1.56 mg/mL. Seventeen phenolic compounds were identified and quantified, with rutin and chlorogenic acid being the most abundant. These findings highlight the plant's potential as a source of bioactive compounds for food applications given its richness in antioxidant and antibacterial agents. However, further in vivo studies are needed to confirm these results and explore its integration into functional foods.

## Author Contributions

**Maroua Hadji**: data curation, investigation, writing – original draft. **Hamdi Bendif**: funding acquisition, project administration, resources, writing – review and editing. **Toka Hadji**: methodology, formal analysis. **Khadidja Dehimi**: visualization, software. **Tahar Smaili**: validation. **Kebaili Fethi Farouk**: methodology. **Ilyas Yildiz**: software, investigation. **Mohamed A. M. Ali**: resources. **Ramazan Erenler**: conceptualization, software, validation. **Amal Lahouaou**: data curation, validation. **Fehmi Boufahja**: funding acquisition, project administration. **Stefania Garzoli**: writing – review and editing, supervision.

## Conflicts of Interest

The authors declare no conflicts of interest.

## Data Availability

The corresponding author will provide all the data in the article upon request, subject to reasonable conditions.
